# Food assistance programs serve the majority of low-income, homebound older adults, yet food insecurity remains

**DOI:** 10.1093/haschl/qxag260

**Published:** 2026-10-01

**Authors:** Yiqing Qian, Mary Louise Pomeroy, Kaitlyn Tang, Laura J Samuel, Kali S Thomas, Katherine A Ornstein

**Affiliations:** Johns Hopkins University School of Nursing Center for Equity in Aging, Baltimore, MD 21205, United States; Johns Hopkins University School of Nursing, Baltimore, MD 21205, United States; University of Arizona College of Medicine—Tucson, Tucson, AZ 85724, United States; Johns Hopkins University School of Nursing, Baltimore, MD 21205, United States; Johns Hopkins University School of Nursing, Baltimore, MD 21205, United States; Johns Hopkins University School of Nursing Center for Equity in Aging, Baltimore, MD 21205, United States

**Keywords:** homebound, food insecurity, food and nutrition service, meals on wheels, home-delivered meals, SNAP

## Abstract

**Introduction:**

Low-income homebound older adults face a disproportionately high risk of food insecurity, yet national evidence of food assistance program uptake among this population is limited.

**Methods:**

Using nationally representative data of Medicare beneficiaries aged 65 and older from the National Health and Aging Trends Study Round 13, we examined Supplemental Nutrition Assistance Program (SNAP) and Meals on Wheels (MOW) participation among 1091 low-income community-dwelling older adults who were homebound and income-eligible for SNAP.

**Results:**

More than half (55.5%) of low-income homebound older adults, representing 2.18 million nationally, participated in at least one program: 35.1% received SNAP only, 10.9% received MOW only, and only 9.4% received both. Despite receiving food assistance, 36.4% still reported food insecurity. We identified 1.75 million low-income homebound older adults who did not receive SNAP or MOW (non-recipients), among whom 24.0% experienced food insecurity and 21.8% lived alone. Among non-recipients, those experiencing food insecurity reported significantly more functional dependencies and were more likely to live alone than those who were food secure.

**Conclusion:**

Policies that expand program capacity, strengthen screening for food insecurity, and streamline enrollment could improve access for this high-need population.

## Introduction

Millions of community-dwelling older adults in the United States are homebound, ie, never or rarely leave the home or require assistance to do so.^1^ Homebound older adults are characterized as having high levels of comorbidity, dementia, functional impairment, lower income, and being more likely to live alone than non-homebound older adults.^1^ Not surprisingly, homebound older adults are more likely to experience food insecurity, ie, limited or uncertain availability of nutritionally adequate and safe foods,^2^ than non-homebound older adults.^3^ Food insecurity coupled with comorbidities can create a negative feedback loop, exacerbating poor health among homebound older adults.^4^

Food assistance programs offer critical support to reduce food insecurity^5^ and its adverse health consequences among older adults.^6^ The nation's largest food assistance program, the Supplemental Nutrition Assistance Program (SNAP), provides a safety net for millions of older adults in low-income households. Evidence links SNAP participation to reduced food insecurity and fewer avoidable nursing home stays.^4,7,8^ Meals on Wheels (MOW), a well-known type of home-delivered meal program, delivers meals to older adults in the community through a national network of over 5000 independent community-based programs.^9^ Receipt of home-delivered meals has been linked to reduced food insecurity and loneliness,^10,11^ making these services particularly critical for homebound older adults who live alone.

Although low-income populations experience disproportionately high rates of food insecurity,^12^ only about half of older adults who are eligible for SNAP actually enroll.^13^ Barriers to participation include stigma, cumbersome enrollment processes, and anticipating low benefit amounts.^14^ While income is not an eligibility criterion for MOW, many clients have low incomes.^15^ Funding constraints within Older Americans Act Nutrition Services Program of 1972, the most significant funding source of MOW, has led to gaps in service including waitlists.^16^ Prior research among older adults on MOW waitlists suggests limited program access and higher enrollment delays for those from lower socioeconomic backgrounds.^16^ Moreover, 2025 federal funding cuts and restrictions for SNAP will likely further intensify the unmet food needs of low-income homebound older adults.

Despite the urgency of access to food assistance programs, population-based evidence focused on older adults who are eligible and likely to benefit most from these programs—specifically homebound, low-income older adults—remains limited. Determining SNAP and MOW participation among this population is necessary to assess gaps in food support programs and can inform related federal and state policies. We estimate the prevalence of food assistance (via SNAP and/or MOW) among low-income homebound older adults in the United States and compare the percentage of food insecurity and key characteristics between those who do and do not participate in these programs.

## Methods

### Data and sample

We used Round 13 (May 2023 to April 2024) data from the National Health and Aging Trends Study (NHATS), a nationally representative survey of health-related trends among Medicare beneficiaries aged 65 and older.^17^ The NHATS Round 13 had an unweighted response rate of 60.6% (weighted response rate: 67.2%).^17^ The sample included low-income, homebound older adults. Homebound status was defined as living in the community but never or rarely leaving home, having difficulty leaving the home, or needing assistance to do so.^1^ Low income was determined as household income at or below 130% of the federal poverty line in 2023, consistent with federal eligibility criteria for SNAP. Missing income data were imputed by the NHATS.^18^  Figure S1 shows sample derivation.

### Measures

Receipt of food assistance was categorized as non-recipients of either program, SNAP recipient only, MOW recipient only, or recipient of both SNAP and MOW. Because SNAP tends to be under-reported in survey data,^19^ we classified NHATS participants as SNAP recipients if they responded “yes” to either of the two items, including (1) “There are several state and federal programs that help people in need. In the last year, did you receive help from any of these programs? A. Food stamps (also called the Supplemental Nutrition Assistance Program, or SNAP)”,^8^ and (2) “In the last month, when you shopped for groceries or other personal items, did you pay for them with cash, check, debit card, credit card, or some other way? D. Food stamps.” Informed by prior MOW research using NHATS data,^15,20,21^ we classified NHATS participants as MOW recipients if they (1) responded “yes” to the question, “There are several state and federal programs that help people in need. In the last year, did you receive help from any of these programs? B. Other food assistance such as Meals-on-Wheels,” or (2) reported ever receiving hot meals from MOW by responding “every time”, “most times”, “sometimes”, or “rarely,” instead of “never,” to the question, “In the last month, when you had hot meals, how often were they from Meals on Wheels or other food assistance programs?”

Food insecurity was measured using a five-item validated measure capturing financial, functional, and social barriers to food access.^3^ The items assess (1) skipping meals due to financial constraints in the last month, (2) skipping meals for greater than a few days, (3) going without groceries in the last month due to lack of ability or social support to do so, (4) going without hot meals in the last month due to lack of ability or social support to do so, and (5) going without eating due to lack of ability or social support to do so. The sum scores range from 0 to 5, where a score of 1 or higher classified respondents as food insecure.

Respondent characteristics included socio-demographics (age, sex, race/ethnicity, education, and rurality), functional dependencies (number of activities of daily living (ADLs) for which they reported difficulty and/or received assistance and number of instrumental activities of daily living (IADLs) for which they received assistance for health or functional reasons), clinical characteristics (self- or proxy-reported hospital stay in the last year and dementia status),^22^ and social network (living alone status, number of living children, and number of family caregivers). Detailed definitions of food insecurity, ADLs, IADLs, and dementia status measures are provided in Table S1.

### Analytic approach

We compared respondents’ food insecurity and other characteristics across food assistance receipt status using chi-square tests and t tests. Analyses applied analytic weights to account for differential probabilities of being selected into NHATS sample and potential nonresponse biases and incorporated NHATS’ complex survey design, including stratification and clustering.^17,23^ Analyses were conducted in R version 4.5.0. using the survey and srvyr packages.

### Limitations

Our assessments of program use relied on survey data, which may be subject to recall bias and include proxy reports. Second, SNAP participation tends to be underreported in survey-based research because of stigma, recall bias, and complex patterns of receipt (eg, irregular receipt).^24^ We also acknowledge the limitations in the NHATS measures of MOW participation. The survey questions group MOW with other food-assistance programs, preventing us from distinguishing MOW from other forms of food assistance. They also do not specifically ask about delivery of frozen meals. Third, SNAP participation in 2023 may have been higher than in typical years because of pandemic-related emergency allotments that ended in February 2023. Fourth, food insecurity and receipt of MOW and SNAP are measured cross-sectionally, thus limiting causal inference. Finally, we acknowledge that our analysis did not capture other important pathways through which many homebound older adults, particularly those with food insecurity and chronic conditions, may receive food assistance, such as Medicare Advantage supplemental benefits,^25^ Commodity Supplemental Food Program,^26^ and services authorized through Medicaid Section 1115 waivers.^27^

## Results

Among our sample of 1091 low-income homebound older adults, 44.5% (1.75 million) did not receive any food assistance. About 55.5% (2.18 million) received food assistance, including 35.1% (1.38 million) who received only SNAP, 10.9% (428 386) who received only MOW, and 9.4% (369 542) who received both SNAP and MOW (Figure 1).

**Figure 1.**
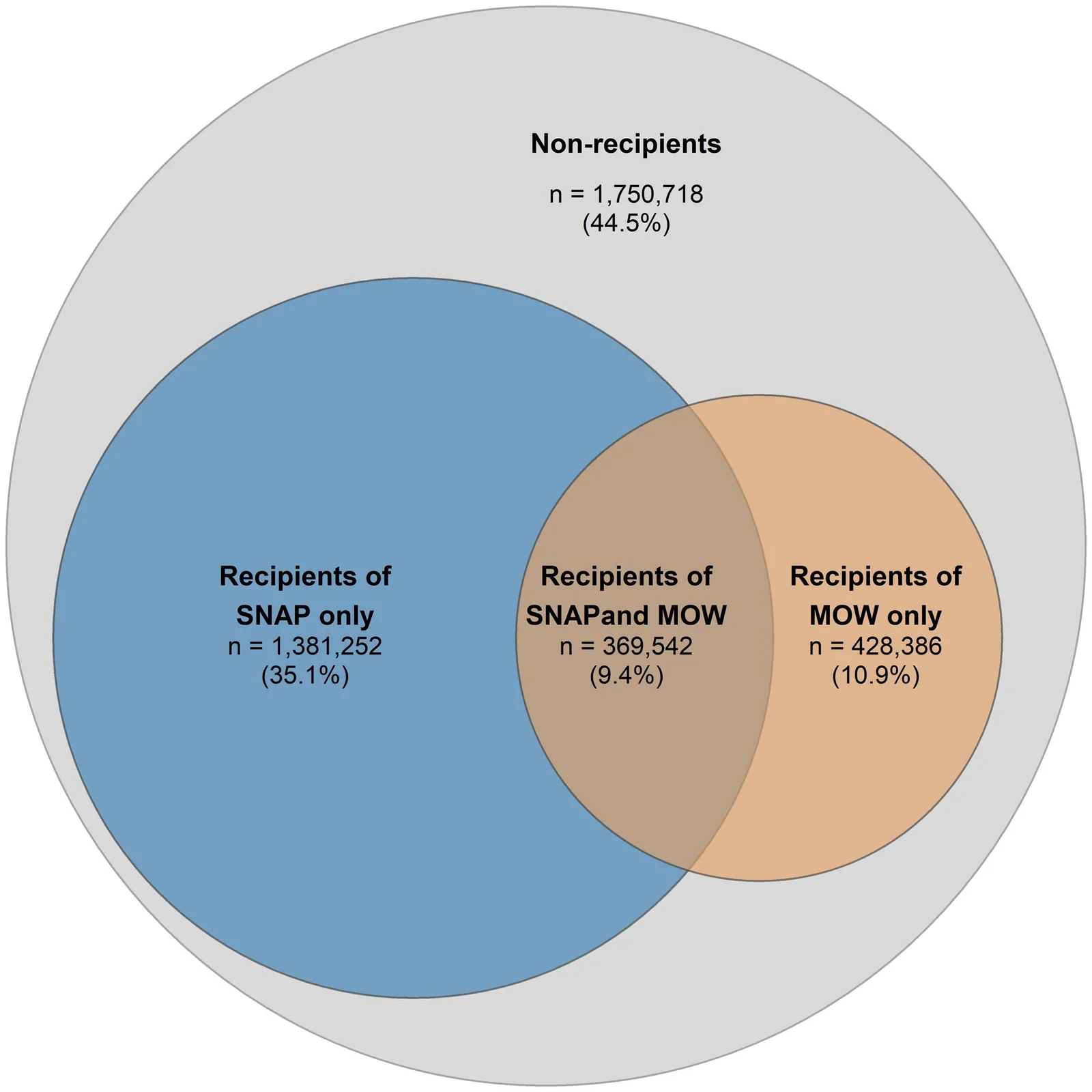
Percentages and overlap in Supplemental Nutrition Assistance Program (SNAP) and Meals on Wheels (MOW) recipients among low-income homebound older adults. Source: Authors’ analysis of data from the National Health and Aging Trends Study Round 13. Notes: The total sample is *n* = 1091 (weighted *N* = 3.9 million), including *n* = 465 non-recipients of SNAP or MOW, *n* = 397 recipients of SNAP only, *n* = 121 recipients of MOW only, and *n* = 108 recipients of both SNAP and MOW. Low income is defined as living in a household at or below 130% of the federal poverty level. SNAP receipt is defined as receiving help from SNAP in the past year and/or using SNAP for grocery shopping in the past month. MOW receipt is defined as receiving help from MOW in the past year and/or receiving hot meals from MOW or other food assistance programs in the past month.

Overall, almost one-third (31.0%) of low-income homebound older adults were food insecure (Table 1). Among those who did not receive food assistance (non-recipients), 24.0% reported food insecurity, lower than those who received food assistance (36.4%, *P* < 0.01). Within non-recipients, those experiencing food insecurity reported significantly more ADL and IADL dependencies and were more likely to live alone than those who were food secure (35.0% vs 17.8%, *P* < 0.05; Table 2 and Table S2). In a sensitivity analysis using a less restrictive definition of SNAP income eligibility (ie, household income below 200% of the federal poverty level, consistent with Broad-Based Categorical Eligibility income criteria for SNAP), we found that 23.0% of non-recipients experienced food insecurity (Table S3). Using a narrower definition of low income (ie, household income 100% below the federal poverty level), we estimated that approximately 22.8% of non-recipients experienced food insecurity (Table S4).

**Table 1.** Food insecurity and other characteristics of low-income homebound older adults with and without food assistance (*N* = 1091).

	Total sample	Receipt status of food assistance		
Characteristics	*N* = 1091	Without food assistance (*n* = 465)	With food assistance (*n* = 626)	*P* value
**Food insecure** (%)	31.0%	24.0%	36.4%	0.009
***Sociodemographic***				
Age (mean, 95%CI)	77.73 (77.10-78.37)	78.33 (77.11-79.55)	77.25 (76.34-78.17)	0.210
Female (%)	72.0%	73.0%	71.4%	0.685
Race and ethnicity (%)				0.092
White, Non-Hispanic	48.2%	53.7%	43.7%	
Black, Non-Hispanic	17.3%	12.7%	20.9%	
Other, Non-Hispanic	7.0%	7.3%	6.8%	
Hispanic	27.5%	26.2%	28.6%	
High school and above (%)	58.4%	62.8%	54.8%	0.027
Rural (%)	17.6%	17.4%	17.8%	0.884
***Functional and clinical***				
ADL dependencies (mean, 95%CI)	4.00 (3.80-4.20)	3.66 (3.32-4.00)	4.27 (4.04-4.50)	0.005
IADL dependencies (mean, 95%CI)	2.10 (1.93-2.26)	1.80 (1.52-2.08)	2.34 (2.13-2.55)	0.005
Probable dementia (%)	26.4%	24.0%	28.4%	0.244
Hospitalization (%)	35.2%	32.8%	37.2%	0.310
***Social support network***				
Living alone (%)	32.8%	21.8%	41.8%	<0.001
Living children (mean, 95%CI)	2.70 (2.54-2.86)	2.62 (2.34-2.91)	2.76 (2.55-2.97)	0.465
Family caregivers (mean, 95%CI)	2.12 (1.97-2.27)	2.16 (1.97-2.36)	2.08 (1.90-2.27)	0.486

Source: Authors’ analysis of data from the National Health and Aging Trends Study (NHATS), Round 13.

Notes: Low-income is defined as living at or below 130% of the federal poverty line. Receipt of food assistance programs is defined as receiving either Supplemental Nutrition Assistance Program (SNAP) and/or Meals on Wheels (MOW). SNAP receipt is defined as receiving help from SNAP in the past year and/or using SNAP for grocery shopping in the past month. MOW receipt is defined as receiving help from MOW in the past year and/or receiving hot meals from MOW or other food assistance programs in the past month. *P* values are from t tests or chi-square tests.

ADLs, activities of daily living; CI, confidence interval; IADLs, instrumental activities of daily living.

**Table 2.** Characteristics of low-income homebound older adults without food assistance by food insecurity status (*N* = 465).

Characteristics	Food secure (*n* = 368)	Food insecure (*n* = 97)	*P* value
***Sociodemographic***			
Age (mean, 95%CI)	78.44 (77.06-79.81)	78.00 (75.60-80.40)	0.746
Female (%)	72.0%	76.0%	0.633
Race and ethnicity (%)			0.298
White, Non-Hispanic	50.5%	64.0%	
Black, Non-Hispanic	14.1%	8.4%	
Other, Non-Hispanic	8.2%	4.3%	
Hispanic	27.2%	23.3%	
High school and above (%)	61.8%	65.4%	0.643
Rural (%)	18.0%	15.4%	0.664
***Functional and clinical***			
ADL dependencies (mean, 95%CI)	3.23 (2.87-3.60)	5.03 (4.41-5.65)	<0.001
IADL dependencies (mean, 95%CI)	1.63 (1.33-1.94)	2.33 (1.71-2.94)	0.049
Probable dementia (%)	24.0%	23.6%	0.951
Hospitalization (%)	30.4%	40.6%	0.226
***Social support network***			
Living alone (%)	17.8%	35.0%	0.049
# Living children (mean, 95%CI)	2.72 (2.44-3.01)	2.31 (1.53-3.09)	0.327
# Family caregivers (mean, 95%CI)	2.11 (1.89-2.33)	2.34 (1.92-2.75)	0.334

Source: Authors’ analysis of data from the National Health and Aging Trends Study (NHATS), Round 13.

Notes: Low-income is defined as living at or below 130% of the federal poverty line. No receipt of food assistance programs is defined as not receiving either Supplemental Nutrition Assistance Program (SNAP) or Meals on Wheels (MOW). SNAP receipt is defined as receiving help from SNAP in the past year and/or using SNAP for grocery shopping in the past month. MOW receipt is defined as receiving help from MOW in the past year and/or receiving hot meals from MOW or other food assistance programs in the past month. *P* values are from t tests or χ^2^ tests.

ADLs, activities of daily living; CI, confidence interval; IADLs, instrumental activities of daily living.

Importantly, food assistance recipients were twice as likely to live alone compared to non-recipients (41.8% vs 21.8%, *P* < 0.01). Within recipients, the proportion living alone was similar between those with food insecurity and those who were food secure (43.4% vs 40.8%, *P* = 0.649; Table S2). Additionally, compared with nonrecipients, food assistance recipients reported more ADL (mean: 4.27 vs 3.66, *P* < 0.01) and IADL dependencies (mean: 2.34 vs 1.80, *P* < 0.01) and were less likely to have a high school education (54.8% vs 62.8%, *P* < 0.05). There were no significant group differences by dementia status, or any other sociodemographic or social support characteristics (Table 1).

Given the limited overlap in MOW and SNAP receipt (Figure 1), we further explored differences in program participation by comparing food insecurity and other characteristics (Table S5) across four food assistance receipt categories (non-recipients, recipients of MOW only, recipients of SNAP only, and recipients of SNAP and MOW). As shown in Figure 2, food insecurity was most prevalent among those who received both programs (44.1%), followed by those who received MOW only (39.4%) and those who received SNAP only (33.6%). We detected significant differences in demographic characteristics by program receipt type. About 45.4% of recipients of SNAP only and 45.8% of recipients of both programs were living alone, a higher proportion than among those receiving MOW only (26.6%). About 61.5% of recipients of SNAP only and 60.3% of recipients of both programs identified as non-White or Hispanic, a higher proportion than among recipients of MOW only (36.0%) (Table S5).

**Figure 2.**
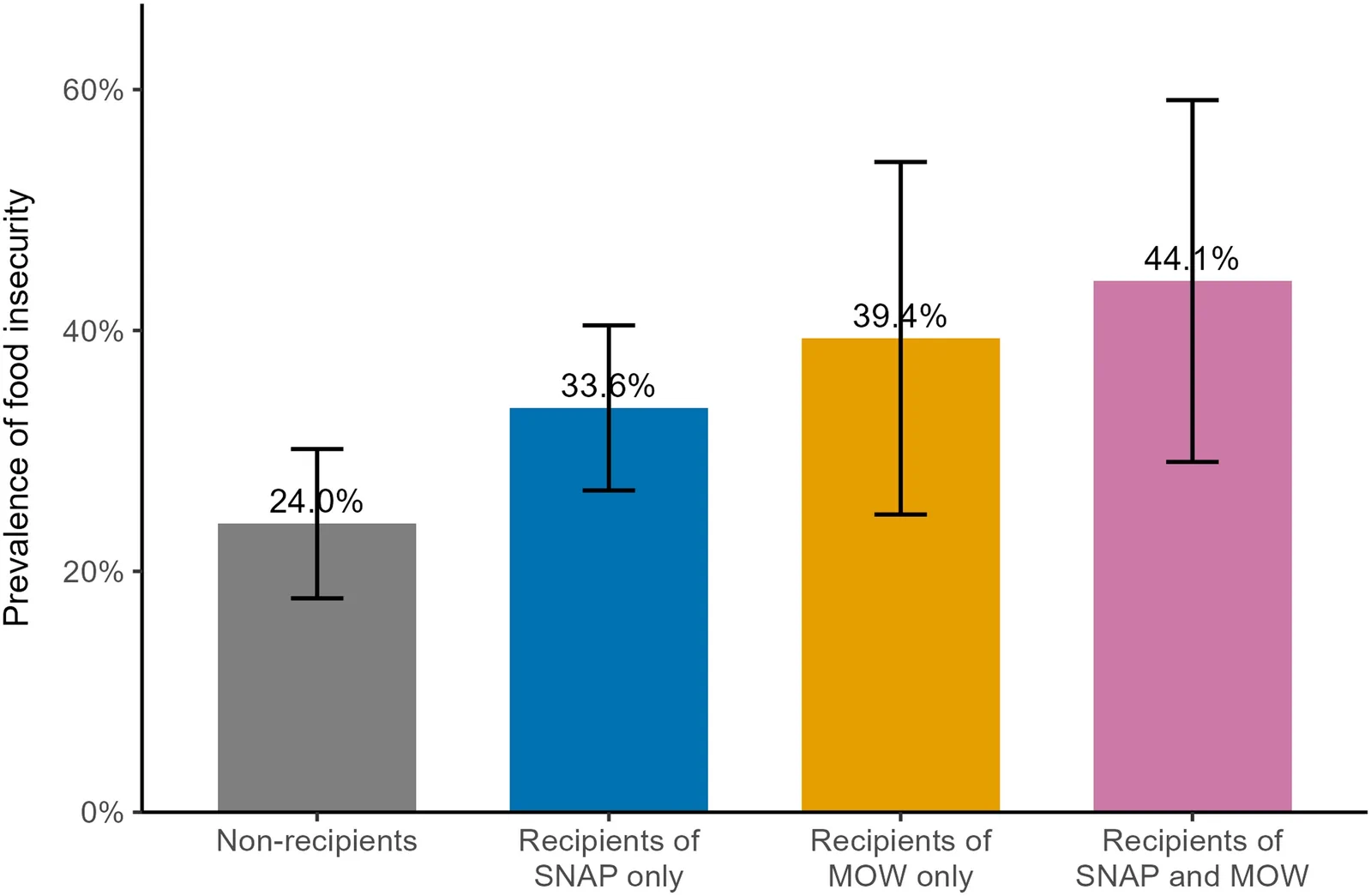
Prevalence of food insecurity among low-income homebound older adults by mutually exclusive categories of supplemental nutrition assistance program (SNAP) and meals on wheels (MOW) receipt. Source: Authors’ analysis of data from the National Health and Aging Trends Study Round 13. Notes: Low-income is defined as living at or below 130% federal poverty line. Abbreviations: SNAP = Supplemental Nutrition Assistance Program. MOW = Meals on Wheels. SNAP receipt is defined as receiving help from SNAP in the past year and/or using SNAP for grocery shopping in the past month. MOW receipt is defined as receiving help from MOW in the past year and/or receiving hot meals from MOW or other food assistance programs in the past month.

## Discussion

Among a national sample of low-income homebound older adults, almost one-third were food insecure. We identified that about 2.18 million (55.5%) received food assistance from either MOW or SNAP. Our finding is consistent with previous research documenting the high prevalence of food insecurity and unique barriers to food access among homebound older adults.^3,28^

We found that low-income homebound older adults who received food assistance had more ADL and IADL dependencies than those who did not receive any food assistance. Using a multi-dimensional measure that captures financial, functional, and social barriers to food access,^3^ we found that food insecurity was also more prevalent among recipients of food assistance programs, particularly those who participated in both programs, than non-recipients. Unlike the traditional focus on financial barriers to food access, such as the U.S. Department of Agriculture (USDA) Household Food Security Survey Module scale,^29^ our estimates of food insecurity reflect a broader conceptualization that captures food access barriers related to functional dependencies and limited social support.^3^ This multi-dimensional measure is particularly relevant to low-income homebound older adults, whose high ADL and IADL dependencies, comorbidity, and reliance on assistance from caregivers, may impede their food access. Despite substantial differences in their eligibility criteria and delivery models, we found that both SNAP and MOW served homebound older adults with high levels of functional limitations and food insecurity.

Although less prevalent among non-recipients, food insecurity remained a substantial issue impacting over one in five low-income homebound older adults, highlighting the unmet food and nutritional needs among this population.^30^ The mechanisms that may inhibit older adults experiencing food insecurity may differ slightly between SNAP and MOW participation. As enrollment and recertification processes for SNAP are often cumbersome, it may not be accessible for individuals with disabilities, including many homebound older adults. In addition to the participation barriers such as stigma of SNAP participation, other administrative burdens associated with SNAP^31^ may particularly impact the homebound population, including learning costs related to understanding eligibility and application procedures and compliance costs associated with documentation and recertification. As homebound older adults may require help with the enrollment process, more flexible policies and supportive services may reduce participation barriers to SNAP. Accessible and simplified enrollment processes, navigation assistance, and coordinated programs may benefit homebound older adults. States can implement flexibilities for administering SNAP that have been shown to increase enrollment among eligible older adults such as the Broad-based Categorical Eligibility with higher income limits,^32^ the Combined Application Project which streamline SNAP enrollment for eligible households receiving Supplemental Security Income,^33^ as well as the Elderly Simplified Application Project which streamlines SNAP application and certification process for eligible households with older adults.^34^

While administrative requirements are less likely a barrier for MOW, constrained access could be. One in three MOW providers reported having a waitlist in 2026 for home-delivered meals.^35^ The growing needs for food support services among older adults, coupled with state and federal funding constraints, contribute to MOW program waitlists and longer enrollment delays, particularly impacting older adults from lower socioeconomic backgrounds.^16^ According to prior research, funding constraints of MOW may also limit programs’ capacity for targeted community outreach, such as to homebound populations.^36,37^ Therefore, additional resources for MOW program are likely to expand the reach and delivery of MOW to homebound older adults experiencing food insecurity.^36^

We also observed a higher proportion of food assistance recipients, particularly SNAP recipients, who lived alone compared with non-recipients, suggesting that these programs may provide critical support for homebound older adults who lack social support within the household. In exploratory multivariable analysis, we found that living alone remained a significant predictor of food assistance receipt even after adjusting for other covariates (Table S6). While SNAP provides greater autonomy in food choices, realizing this benefit requires that homebound older adults have capacity or support to buy and prepare food. On the other hand, MOW programs are more directly tailored to the needs of homebound older adults by delivering prepared meals, and may have the added benefits of promoting social connection through driver interactions and providing assistance with household or personal tasks (eg, getting the mail).^10,11^ Moreover, although non-recipients overall were less likely to live alone than recipients, we found about 35% of food insecure non-recipients were living alone, almost twice the prevalence of food-secure non-recipients. Future programs should explore strategies to better reach and assist homebound older adults living alone in enrolling in MOW and SNAP, and to bolster transportation assistance as a means to facilitate SNAP use and enhance food choice.

Our study points to the potential of better integrating health care and social services to address complex care needs of homebound older adults. As recommended by the Centers for Medicare and Medicaid Services,^38^ implementing standardized screening for food insecurity and other health-related social needs (eg, transportation needs) in clinical and home-based care settings may serve as a critical pathway to connecting homebound older adults with resources and services in the community. The high prevalence of food insecurity among non-recipients suggests the need for screening and timely referrals during routine and urgent medical visits, and through home-based clinical or support services for homebound older adults. Since homebound status among older adults can be dynamic,^39^ routine assessments as well as timely referral and receipt of services are essential. For instance, receipt of MOW can be particularly beneficial for older adults who are newly homebound following hospital discharge, helping them remain safely at home and prevent further illness exacerbations.^40^ Promoting partnerships between health care systems and community-based services,^41^ such as MOW, are critical to optimize care and support this high-need population.

## Conclusions

This nationally representative study documents that food assistance programs, including SNAP and MOW, serve more than half of U.S. low-income homebound older adults. Those with higher care and food needs and limited social support are more likely to receive these services. Yet, gaps in access remain and will likely widen due to chronic underfunding of MOW and recent federal funding cuts to SNAP, potentially causing substantial harm to this growing population with significant unmet needs.^30^ Amid rising food costs due to inflation and increasing rates of food insecurity among older adults,^9,42^ sustained federal funding for SNAP and MOW is both essential and urgent. Strengthening routine assessments of unmet needs, timely referrals, and enrollment support for community services, including food assistance programs, is critical to helping this population maintain food security and independent living in the community.

## Supplementary Material

qxag260_Supplementary_Data
