# Supplementary material for: Food assistance programs serve the majority of low-income, homebound older adults, yet food insecurity remains
Source: Health Aff Sch. 2026 Oct 1;4(10):qxag260. doi: 10.1093/haschl/qxag260 (PMC13648203; doi:10.1093/haschl/qxag260)
Supplement: Supplementary file 1 [file qxag260_supplementary_data.zip › homebound food assistance_appendix_09182026.docx]

**Supplementary Material**

**Supplementary Figure 1**. **Consort diagram of sample selection**

**Source:** National Health and Aging Trends Study (NHATS) Round 13.

**Supplementary Table 1. Definition of measures of food insecurity, activities of daily living (ADLs), instrumental activities of daily living (IADLs), and dementia status**

| **Variable** | **Definition** |
| --- | --- |
| Food insecurity | Food insecurity was measured using a five-item summary indicator including a) skipping meals due to financial constraints in the last month, b) skipping meals for greater than a few days, c) going without groceries in the last month due to lack of ability or social support to do so, d) going without hot meals in the last month due to lack of ability or social support to do so, and e) going without eating due to lack of ability or social support to do so. Sum scores ranged from 0–5, where a score of 1 or higher was classified as food insecure. |
| Dependency in activities of daily living (ADLs) | ADL dependencies was measured as the number of ADLs for which respondents received assistance was based on seven activities: eating, bathing, toileting, getting out of bed, getting around inside, and getting around outside. |
| Dependency in instrumental activities of daily living (IADLs) | IADL dependencies was measured as the number of IADLs which respondents received assistance for health or functional reasons was based on five activities: laundry, shopping, meal preparation, medication management, and bills/banking. |
| Dementia status | Respondents' probable dementia status was determined using the validated NHATS dementia classification algorithm. The algorithm incorporates three sources of information: (1) self- or proxy-report of a physician diagnosis of dementia or Alzheimer's disease; (2) proxy responses to the 8-item AD8 Dementia Screening Interview, which assesses memory, temporal orientation, judgment, and functional abilities; and (3) cognitive testing of memory, orientation, and executive function. The algorithm classifies respondents as having no dementia, possible dementia, or probable dementia. In alignment with prior work, respondents with no dementia and possible dementia were combined into a single no dementia category. |

**Notes:** Measures were consistent with prior work using the National Health and Aging Trends Study (NHATS). Food insecurity was measured using the validated NHATS summary indicator: Tucher EL, Keeney T, Cohen AJ, Thomas KS. Conceptualizing Food Insecurity Among Older Adults: Development of a Summary Indicator in the National Health and Aging Trends Study. J Gerontol B Psychol Sci Soc Sci. 2021;76(10):2063-72. Dementia status was classified using the NHATS dementia classification algorithm: Kasper JD, Freedman VA, Spillman B. Classification of Persons by Dementia Status in the National Health and Aging Trends Study. Technical Paper #5. 2013.

**Supplementary Table 2. Characteristics of low-income homebound older adults with and without food assistance by food insecurity status (N=1,091)**

|  | **Without food assistance**  **(n=465)** | | | **With food assistance**  **(n=626)** | | |
| --- | --- | --- | --- | --- | --- | --- |
| Characteristics | Food secure (*n*=368) | Food insecure (*n*=97) | *P* value | Food secure (*n*=422) | Food insecure (*n*=204) | *P* value |
| ***Sociodemographic*** |  |  |  |  |  |  |
| Age (mean, 95%CI) | 78.44 (77.06–79.81) | 78.00 (75.60–80.40) | 0.746 | 77.41 (76.31–78.51) | 76.99 (75.38–78.59) | 0.665 |
| Female (%) | 72.0% | 76.0% | 0.633 | 71.4% | 71.0% | 0.952 |
| Race and ethnicity (%) |  |  | 0.298 |  |  | 0.375 |
| White, Non-Hispanic | 50.5% | 64.0% |  | 43.2% | 44.6% |  |
| Black, Non-Hispanic | 14.1% | 8.4% |  | 22.9% | 17.3% |  |
| Other, Non-Hispanic | 8.2% | 4.3% |  | 5.6% | 9.0% |  |
| Hispanic | 27.2% | 23.3% |  | 28.3% | 29.1% |  |
| High school and above (%) | 61.8% | 65.4% | 0.643 | 52.8% | 58.2% | 0.394 |
| Rural (%) | 18.0% | 15.4% | 0.664 | 19.4% | 15.2% | 0.279 |
| ***Functional and clinical*** |  |  |  |  |  |  |
| # ADL dependencies (mean, 95%CI) | 3.23 (2.87–3.60) | 5.03 (4.41–5.65) | <0.001 | 3.72 (3.46–3.98) | 5.22 (4.89–5.55) | <0.001 |
| # IADL dependencies (mean, 95%CI) | 1.63 (1.33–1.94) | 2.33 (1.71–2.94) | 0.049 | 2.19 (1.95–2.44) | 2.60 (2.22–2.97) | 0.074 |
| Probable dementia (%) | 24.0% | 23.6% | 0.951 | 28.6% | 28.2% | 0.934 |
| Hospitalization (%) | 30.4% | 40.6% | 0.226 | 31.6% | 46.8% | 0.009 |
| ***Social support network*** |  |  |  |  |  |  |
| Living alone (%) | 17.8% | 35.0% | 0.049 | 40.8% | 43.4% | 0.649 |
| # Living children (mean, 95%CI) | 2.72 (2.44–3.01) | 2.31 (1.53–3.09) | 0.327 | 2.78 (2.53–3.02) | 2.74 (2.39–3.08) | 0.846 |
| # Family caregivers (mean, 95%CI) | 2.11 (1.89–2.33) | 2.34 (1.92–2.75) | 0.334 | 2.01 (1.79–2.23) | 2.21 (1.89–2.54) | 0.297 |

**Source*:*** Authors’ analysis of data from the National Health and Aging Trends Study (NHATS), Round 13. **Notes*:*** Low-income is defined as living at or below 130% of the federal poverty line. Receipt of food assistance programs is defined as receiving either Supplemental Nutrition Assistance Program (SNAP) and/or Meals on Wheels (MOW). SNAP receipt is defined as receiving help from SNAP in the past year and/or using SNAP for grocery shopping in the past month. MOW receipt is defined as receiving help from MOW in the past year and/or receiving hot meals from MOW or other food assistance programs in the past month. P values are from t tests or chi-square tests. ADLs=Activities of daily living. IADLs=Instrumental activities of daily living. CI=Confidence Interval.

**Supplementary Table 3. Food insecurity and other characteristics by food assistance receipt status among homebound older adults living below 200% federal poverty level (N=1,513)**

|  | **Receipt status of food assistance** | | |
| --- | --- | --- | --- |
| Characteristics | ***Without food assistance***  Unweighted n=778  Weighted n=3,211,884 | ***With food assistance***  Unweighted n=735 Weighted n=2,740,355 | P value |
| **Food insecure** (%) | 23.0% | 36.0% | 0.007 |
| ***Sociodemographic*** |  |  |  |
| Age (mean, 95%CI) | 78.17 (77.18–79.16) | 77.28 (76.24–78.31) | 0.289 |
| Female (%) | 68.8% | 69.2% | 0.921 |
| Race and ethnicity (%) |  |  | 0.002 |
| White, Non-Hispanic | 62.5% | 46.5% |  |
| Black, Non-Hispanic | 12.3% | 19.7% |  |
| Other, Non-Hispanic | 6.2% | 7.5% |  |
| Hispanic | 19.0% | 26.3% |  |
| High school and above (%) | 71.2% | 58.0% | <0.001 |
| Rural (%) | 19.4% | 17.4% | 0.522 |
| ***Functional and clinical*** |  |  |  |
| # ADL dependencies (mean, 95%CI) | 3.60 (3.37–3.84) | 4.23 (4.03–4.44) | <0.001 |
| # IADL dependencies (mean, 95%CI) | 1.71 (1.50–1.92) | 2.35 (2.14–2.55) | <0.001 |
| Probable dementia (%) | 24.6% | 30.0% | 0.090 |
| Hospitalization (%) | 34.0% | 38.0% | 0.234 |
| ***Social support network*** |  |  |  |
| Living alone (%) | 29.2% | 45.0% | <0.001 |
| # Living children (mean, 95%CI) | 2.60 (2.39–2.80) | 2.66 (2.47–2.85) | 0.691 |
| # Family caregivers (mean, 95%CI) | 2.02 (1.89–2.15) | 2.13 (1.98–2.28) | 0.244 |

**Source:** Authors’ analysis of survey data from National Health and Aging Trends Study, Round 13. **Notes:** Receipt of food assistance is defined as receiving either Supplemental Nutrition Assistance Program and/or Meals on Wheels. P values are from t tests or chi-square tests. ADLs=Activities of daily living. IADLs=Instrumental activities of daily living. CI=Confidence Interval.

**Supplementary Table 4. Food insecurity and other characteristics by food assistance receipt status among homebound older adults living below federal poverty level (N=888)**

|  | **Receipt status of food assistance** | | |
| --- | --- | --- | --- |
| Characteristics | ***Without food assistance***  Unweighted n=348  Weighted n=1,252,055 | ***With food assistance***  Unweighted n=541 Weighted n=1,845,866 | P value |
| **Food insecure** (%) | 22.8% | 35.4% | 0.012 |
| ***Sociodemographic*** |  |  |  |
| Age (mean, 95%CI) | 77.91 (76.5–79.31) | 77.26 (76.29–78.24) | 0.478 |
| Female (%) | 76.6% | 73.6% | 0.585 |
| Race and ethnicity (%) |  |  | 0.299 |
| White, Non-Hispanic | 47.6% | 39.7% |  |
| Black, Non-Hispanic | 13.2% | 21.2% |  |
| Other, Non-Hispanic | 8.1% | 7.9% |  |
| Hispanic | 31.1% | 31.2% |  |
| High school and above (%) | 57.0% | 51.4% | 0.250 |
| Rural (%) | 18.0% | 17.4% | 0.897 |
| ***Functional and clinical*** |  |  |  |
| # ADL dependencies (mean, 95%CI) | 3.54 (3.10–3.98) | 4.24 (3.97–4.52) | 0.006 |
| # IADL dependencies (mean, 95%CI) | 1.83 (1.50–2.17) | 2.32 (2.12–2.53) | 0.0192 |
| Probable dementia (%) | 25.4% | 29.4% | 0.347 |
| Hospitalization (%) | 33.4% | 32.4% | 0.841 |
| ***Social support network*** |  |  |  |
| Living alone (%) | 23.4% | 42.0% | <0.001 |
| # Living children (mean, 95%CI) | 2.60 (2.24–2.96) | 2.85 (2.64–3.07) | 0.238 |
| # Family caregivers (mean, 95%CI) | 2.16 (1.91–2.41) | 2.10 (1.87–2.33) | 0.647 |

**Source:** Authors’ analysis of survey data from the National Health and Aging Trends Study, Round 13. **Notes:** Receipt of food assistance is defined as receiving either Supplemental Nutrition Assistance Program and/or Meals on Wheels. P values are from t tests or chi-square tests. ADLs=Activities of daily living. IADLs=Instrumental activities of daily living. CI=Confidence Interval.

**Supplementary Table 5.** **Food insecurity and other characteristics by recipient status of SNAP and MOW among low-income homebound older adults (N=1,091)**

| Characteristics | **Non-recipient**  **(n=465)** | **Recipient of SNAP only**  **(n=397)** | **Recipient of MOW only**  **(n=121)** | **Recipient of both SNAP and MOW (n=108)** | P value |
| --- | --- | --- | --- | --- | --- |
| **Food insecure** (%) | 24.0% | 33.6% | 39.4% | 44.2% | 0.028 |
| ***Sociodemographic*** |  |  |  |  |  |
| Age (mean, 95%CI) | 78.33 (77.11–79.55) | 77.23 (76.13–78.32) | 79.40 (77.25–81.56) | 74.87 (73.33–76.40) | 0.001 |
| Female (%) | 73.0% | 76.0% | 64.0% | 62.0% | 0.197 |
| Race and ethnicity (%) |  |  |  |  | 0.009 |
| White, Non-Hispanic | 53.7% | 38.5% | 64.0% | 39.7% |  |
| Black, Non-Hispanic | 12.7% | 22.0% | 14.9% | 23.4% |  |
| Other, Non-Hispanic | 7.3% | 9.5% | 1.3% | 3.3% |  |
| Hispanic | 26.2% | 30.0% | 19.8% | 33.5% |  |
| High school and above (%) | 62.8% | 51.6% | 61.2% | 59.6% | 0.115 |
| Rural (%) | 17.4% | 16.8% | 20.6% | 18.8% | 0.920 |
| ***Functional and clinical*** |  |  |  |  |  |
| # ADL dependencies (mean, 95%CI) | 3.66 (3.32–4.00) | 4.19 (3.89–4.49) | 4.40 (3.93–4.88) | 4.41 (3.71–5.11) | 0.042 |
| # IADL dependencies (mean, 95%CI) | 1.80 (1.52–2.08) | 2.24 (1.96–2.52) | 2.41 (1.95–2.88) | 2.61 (2.08–3.14) | 0.028 |
| Probable dementia (%) | 24.0% | 27.6% | 37.0% | 21.8% | 0.162 |
| Hospitalization (%) | 32.8% | 31.8% | 48.6% | 44.2% | 0.100 |
| ***Social support network*** |  |  |  |  |  |
| Living alone (%) | 21.8% | 45.4% | 26.6% | 45.8% | <0.001 |
| # Living children (mean, 95%CI) | 2.62 (2.34–2.91) | 2.79 (2.55–3.03) | 2.72 (2.20–3.24) | 2.70 (2.14–3.27) | 0.829 |
| # Family caregivers (mean, 95%CI) | 2.16 (1.97–2.36) | 2.03 (1.76–2.30) | 2.42 (2.01–2.83) | 1.90 (1.52–2.27) | 0.164 |

**Source:** Authors’ analysis of survey data from the National Health and Aging Trends Study, Round 13. **Notes:** Low-income is defined as living at or below 130% federal poverty line. SNAP=Supplemental Nutrition Assistance Program. MOW=Meals on Wheels. P values are from F tests or chi-square tests. ADLs=Activities of daily living. IADLs=Instrumental activities of daily living. CI=Confidence Interval.

**Supplementary Table 6. Multivariate logistic regression model on receipt status of food assistance among low-income homebound older adults (N=1,091)**

| **Variable** | **OR** | **95% CI** | | **P value** |
| --- | --- | --- | --- | --- |
| Food insecure vs food secure | 1.44 | 0.9 | 2.32 | 0.127 |
| ***Sociodemographic*** |  |  |  |  |
| Age | 0.97 | 0.94 | 1.00 | 0.023 |
| Female vs male | 0.79 | 0.49 | 1.28 | 0.338 |
| Race and ethnicity (ref=White, non-Hispanic) |  |  |  |  |
| Black, Non-Hispanic | 1.79 | 1.11 | 2.91 | 0.019 |
| Other, Non-Hispanic | 1.22 | 0.45 | 3.31 | 0.684 |
| Hispanic | 1.38 | 0.84 | 2.25 | 0.193 |
| High school and above vs below high school | 0.78 | 0.54 | 1.14 | 0.199 |
| Rural vs metro | 1.04 | 0.55 | 1.97 | 0.911 |
| ***Functional and clinical*** |  |  |  |  |
| # ADL dependencies | 1.05 | 0.94 | 1.19 | 0.382 |
| # IADL dependencies | 1.2 | 1.03 | 1.39 | 0.021 |
| Probable dementia vs non-dementia | 1.22 | 0.79 | 1.86 | 0.359 |
| Hospitalization vs no hospitalization | 1.04 | 0.68 | 1.59 | 0.864 |
| ***Social support network*** |  |  |  |  |
| Living alone vs not alone | 3.28 | 2.00 | 5.36 | <0.001 |
| # Living children | 1.06 | 0.96 | 1.18 | 0.260 |
| # Family caregivers | 0.94 | 0.81 | 1.09 | 0.404 |

**Source:** Authors’ analysis of survey data from the National Health and Aging Trends Study, Round 13. **Notes:** Low-income is defined as living at or below 130% federal poverty line. Receipt of food assistance programs is defined as receiving either Supplemental Nutrition Assistance Program and/or Meals on Wheels. ADLs=Activities of daily living. IADLs=Instrumental activities of daily living. OR=Odds Ratio. CI=Confidence Interval.
